# A Study of the Dynamic Relation between Physiological Changes and Spontaneous Expressions

**DOI:** 10.1038/s41598-017-07122-x

**Published:** 2017-08-01

**Authors:** Fenglei Yang, Sijung Hu, Baomin Li, Vincent M. Dwyer, Harnani Hassan, Dong-Qing Wei, Ping Shi

**Affiliations:** 1 Shanghai University, School of Computer Engineering and Science, Shanghai, 200444 China; 2 Loughborough University, Wolfson School of Mechanical, Electrical and Manufacturing Engineering, Loughborough, Leicestershire, LE11 3TU UK; 3 East China Normal University, Faculty of Education, Shanghai, 200062 China; 4 Shanghai Jiaotong University, College of Life Sciences and Biotechnology, Shanghai, 200240 China; 5 University of Shanghai for Science and Technology, Institute of Rehabilitation Engineering and Technology, Shanghai, 200093 China

## Abstract

Recent progress in Affective Computing (AC) has enabled integration of physiological cues and spontaneous expressions to reveal a subject’s emotional state. Due to the lack of an effective technique for evaluating multimodal correlations, experience and intuition play a main role in present AC studies when fusing affective cues or modalities, resulting in unexpected outcomes. This study seeks to demonstrate a dynamic correlation between two such affective cues, physiological changes and spontaneous expressions, which were obtained by a combination of stereo vision based tracking and imaging photoplethysmography (iPPG), with a designed protocol involving 20 healthy subjects. The two cues obtained were sampled into a Statistical Association Space (SAS) to evaluate their dynamic correlation. It is found that the probability densities in the SAS increase as the peaks in two cues are approached. Also the complex form of the high probability density region in the SAS suggests a nonlinear correlation between two cues. Finally the cumulative distribution on the zero time-difference surface is found to be small (<0.047) demonstrating a lack of simultaneity. These results show that the two cues have a close interrelation, that is both asynchronous and nonlinear, in which a peak of one cue heralds a peak in the other.

## Introduction

Researchers in the field of AC believe that equipping a computer with the ability to automatically recognize and respond to a user’s affective state could make the computer interface more usable, enjoyable, and effective^1^. For instance, an affect-sensitive learning environment able to recognize and respond to frustration is expected to increase the motivation for study, and improve learning compared to an affect-insensitive environment. Inspired by this belief, AC research has endeavored to narrow the communicative gap between the highly emotional human and an emotionally challenged computer and a number of affect-sensitive systems have been developed in several domains, including gaming, mental health, and learning technologies^2–6^.

Due to the clarity of the human face when displaying an emotive state, facial expressions are naturally used as an important input to AC systems. Unfortunately, existing studies on facial expressions are largely based on deliberation, and often exaggerated facial displays, irrespective of whether these expressions are relevant to real AC applications^7^. To address this issue, a small number of studies have focused on spontaneous facial expressions^3, 4, 8–12^. Spontaneous facial expressions represent a more honest embodiment of naturally occurring emotions and thus could assist AC systems to better reveal a person’s true affective state.

Recent studies^5–21^ have attempted to integrate physiological changes with facial expressions to improve the reliability of the appraisal of an affective state. A multimodal paradigm is based on the view that an emotional episode could activate both physiological changes and behavioral responses. Anger, for instance, could be manifest via particular facial, vocal, and bodily expressions, together with physiological changes such as increased heart rate, and may be accompanied by other dispute actions. Largely due to the challenge of fusing the heterogeneous information, such a multimodal paradigm is widely advocated, but rarely implemented.

Defining a reliable strategy to fuse physiological changes and spontaneous expressions, which are derived from various sources, on different time scales, with different metric levels and different temporal structures, is a difficult task. Optimal fusion requires an effective evaluation of the temporal dynamic correlations between the two affective cues. However, such fundamental evaluation work has yet to be accomplished^1, 7, 22^. The absence of a reliable method for the evaluation leaves experience and intuition as the predominant role in current AC fusion studies, and this unavoidably results in unexpected outcomes. For instance, the body of work^5, 6, 17, 18^ considered data synchronization to fuse two affective cues that were assumed to be correlated and simultaneous. Yet, the current study shows that, although during an emotional episode, the expression responses and the physiological changes possess a good correlation to each other, there is a high probability that this could occur in an asynchronous manner. In other work^20^, a linear fusion model was used which is again contrary to the objective experimental observation of the current study which shows that the correlations between two cues can have complex (nonlinear) distribution characteristics. To minimize the complexity of fusion, another method^23^ was developed to filter out the time-dependence of affective cues by extracting statistical features such as means, standard deviations (SD), and extreme values. However the resulting reduction in the time-dependent information will undoubtedly reduce the performance of the AC fusion.

Therefore, an effective evaluation of dynamic correlation of affective cues is necessary to achieve optimal fusion for AC. This study seeks to achieve such an effective evaluation by focusing on a temporal dynamic correlation between physiological changes and spontaneous expressions. Data for two affective cues was obtained through a combination of stereo vision based tracking^24^ and imaging photoplethysmography (iPPG)^25^, with a designed experimental protocol including 20 subjects. This represents a practical and easily acceptable approach in a real-world scenario.

Integration of the two technologies is easy to implement in a manner which does not impact on the subjects who are able to express their emotions freely and naturally. Both are non-contact approaches which do not interfere with each other, yet are able to track the spontaneous expressions. The stereo vision used a pair of remote cameras to track precisely 3D landmarks on the face for the spontaneous expression measurement. By utilizing a remote camera, iPPG achieves a long-range extraction of physiological changes from a region of interest (ROI) on the forehead. iPPG is an emerging imaging technology, able to provide some vital human bioinformatics such as heart rate (HR)^26^ and pulse transit time (PTT)^27^. The recent progress on iPPG has demonstrated a significant improvement over conventional PPG, as it removes the primary limitation of spot measurement and sensory contact.

Statistical association space (SAS) extends the usual 2D correlation matrix^28, 29^ by appending the additional dimension of *time-difference* to the points sampled from physiological changes and spontaneous expressions. A non-parametric joint probability density estimation method is usually used in the SAS to model temporal dependencies between the sample points. The utilization of sampled points in modeling enables an objective observation of the instantaneous correlation of two cues, and permits a dynamic correlation evaluation which does not require any priori definition or hypothesis. Moreover, signal intensity changes that are directly calculated from facial motions, rather than manual discrete expression labels or dimensional descriptions^7^, are used to represent the instantaneous expressions. This further ensures the objectivity of sampled points, which are justified by stereo vision^24^, iPPG^26, 27, 30–32^, the Lowess Smoothing algorithm^33, 34^, and a derivative math theory. The outcome shows that the two cues have a close correlation, but in an asynchronous and nonlinear way, as a peak in one cue leads or lags an associated peak in the other.

## Results

### Validation of heart rate measurement

**Figure 1:**
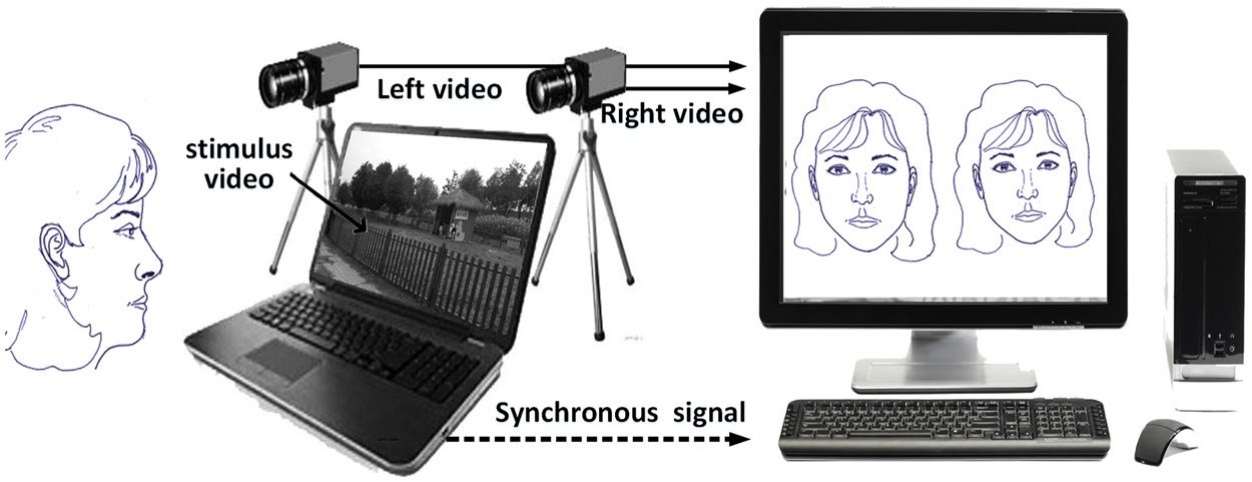
An experimental layout for expression induction, capture and synchronization.

**Figure 2:**
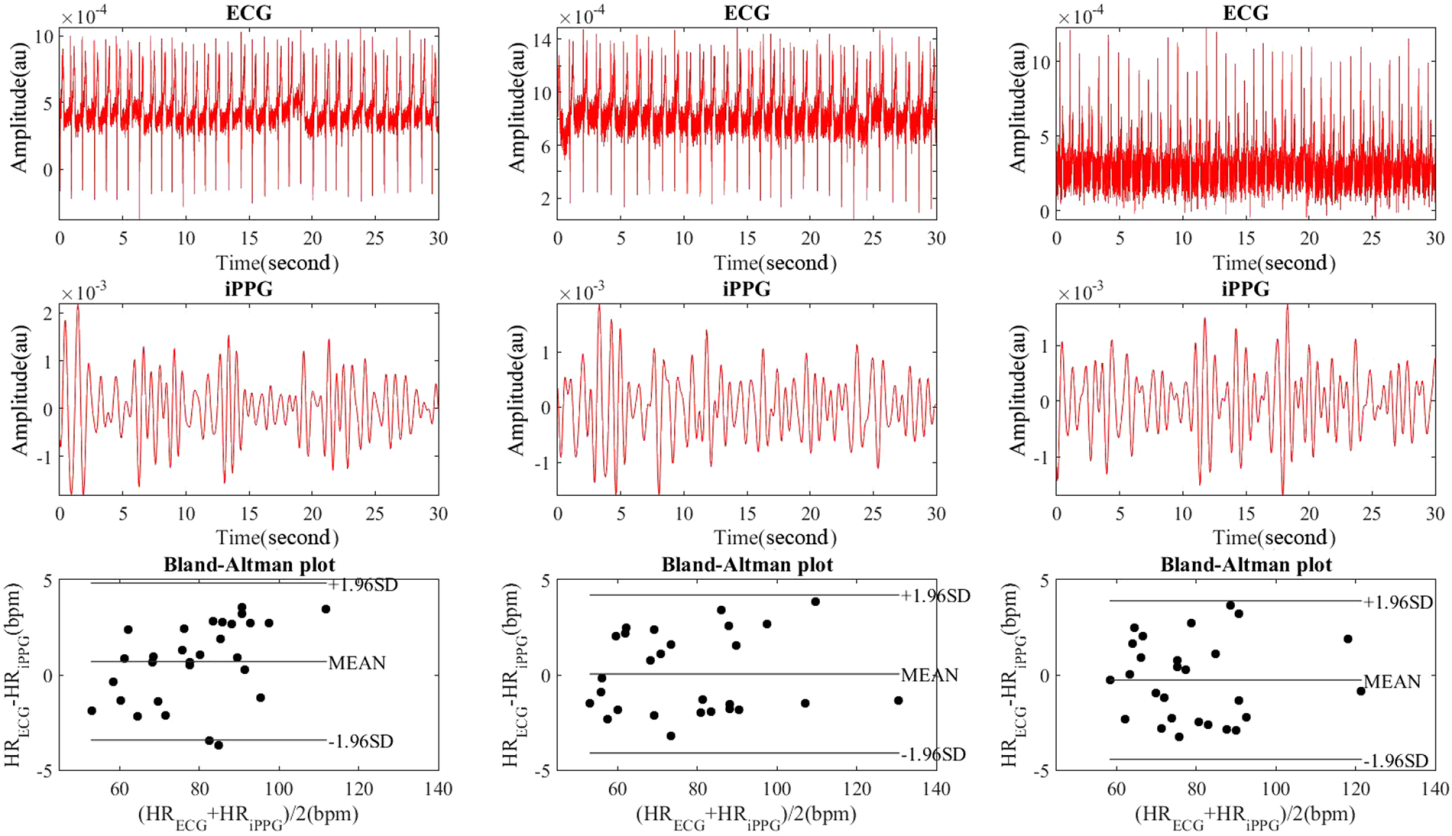
Heart-rate cues and Bland-Altman plots in an initial experiment with three subjects, one per column.

The experimental setup for the current study is shown in Figure 1. Heart Rate (HR) measurements were obtained by iPPG analysis of a single ROI on the subject, while Expression Energy (EE) measurements were obtained from the relative displacement of detectable landmarks. To validate the HR measurement, a Bland-Altman analysis was performed on three subjects (prior to the main experiment and separated from it) to assess the agreement between HR measurements obtained by iPPG and ECG. In Figure 2, the first two rows display the HRs of three subjects, taken by ECG and iPPG respectively, while the final row displays the results of Bland-Altman analysis. The mean difference between the two techniques shows them to be not significantly different. Specifically, the three mean values between the ECG and iPPG signals are 0.69, 0.04, and −0.28 beats per minute (bpm), respectively. The corresponding 95% limits of agreement are from −3.42 to 4.82 bpm, from −4.11 to 4.20 bpm, and from −4.44 to 3.89 bpm. Furthermore, there is a significant correlation of HR obtained by electrocardiogram (ECG) and iPPG for these three subjects (a Pearson’s correlation shows *r* ^2^ > 0.83, *p* < 0.01).

### Measurement of facial expression and heart rate (HR)

The measurement of facial expression was obtained from the positions in a frame of the facial landmarks. The choice of these landmarks was made to focus on the active regions of facial motion, and a total of 66 landmarks (on each face) was detected. This includes: 10 landmarks on the eye-brows; 16 landmarks on the eyes; one landmark midway between the eyes; eight landmarks on the nose; four landmarks on the cheeks; 14 landmarks on the mouth; seven landmarks on the chin and six landmarks on pupils. Recovering the 3D position of the landmarks in a frame, the expression energy was calculated, frame by frame, from Equation (2). The resulting expression energy (EE) series resampled at 10 second intervals are tabulated in Table 1.

**Table 1:** Expression energies of 20 subjects, at 10 second intervals.

Subjects	Time Interval(second)															
	10	20	30	40	50	60	70	80	90	100	110	120	130	140	150	160
1	1.70	1.73	1.74	1.74	1.74	1.80	1.67	1.46	1.87	1.70	1.59	1.57	1.61	1.63	1.69	1.69
2	1.63	1.65	1.64	1.63	1.64	1.62	1.53	1.55	1.60	1.61	1.61	1.61	1.62	1.63	1.67	1.93
3	1.97	2.01	1.91	2.04	1.88	2.00	1.79	1.76	1.70	1.69	1.81	1.84	1.73	1.90	1.95	1.85
4	2.20	2.11	2.06	1.98	1.70	1.77	1.57	1.57	1.64	1.65	1.61	1.57	1.56	1.55	1.70	1.72
5	1.94	1.91	2.02	1.90	1.89	1.86	1.86	1.88	1.79	1.77	1.77	1.73	1.74	1.77	1.76	1.84
6	1.65	1.64	1.63	1.59	1.63	1.63	1.64	1.64	1.52	1.55	1.52	1.59	1.58	1.51	1.54	1.73
7	2.09	1.75	1.69	1.65	1.69	1.85	1.79	1.79	1.67	1.69	1.67	1.66	1.66	1.62	1.72	1.87
8	1.40	1.36	1.37	1.46	1.39	1.43	1.34	1.26	1.26	1.32	1.31	1.29	1.29	1.38	1.24	1.24
9	1.55	1.37	1.49	1.60	1.42	1.62	1.58	1.48	1.38	1.42	1.40	1.43	1.40	1.36	1.36	1.50
10	2.02	1.65	1.57	1.59	1.79	1.68	1.69	1.71	1.54	1.55	1.52	1.49	1.49	1.48	1.46	1.97
11	1.98	1.98	1.69	1.58	1.49	1.56	1.82	1.71	1.50	1.53	1.50	1.52	1.56	1.59	1.62	1.52
12	1.60	1.69	1.77	1.71	1.56	1.74	1.62	1.63	1.73	1.62	1.63	1.55	1.55	1.55	1.77	2.00
13	1.77	1.76	1.76	1.77	1.76	1.85	1.78	1.73	1.75	1.76	1.75	1.76	1.77	1.77	1.76	1.76
14	1.52	1.54	1.54	1.53	1.46	1.51	1.47	1.46	1.49	1.48	1.51	1.46	1.47	1.48	1.50	1.49
15	2.05	1.73	1.69	1.73	1.72	1.61	1.65	1.64	1.60	1.63	1.70	1.67	1.59	1.61	1.70	2.52
16	2.05	1.85	2.01	1.90	1.61	1.65	1.62	1.46	1.45	1.45	1.48	1.48	1.45	1.46	1.47	2.00
17	1.62	1.61	1.61	1.63	1.59	1.62	1.64	1.64	1.59	1.61	1.62	1.62	1.63	1.63	1.64	1.84
18	2.50	2.26	2.08	2.14	2.38	2.45	2.43	2.41	1.98	2.09	2.01	1.94	1.92	1.45	1.54	2.60
19	1.65	1.50	1.51	1.63	1.42	1.59	1.37	1.33	1.33	1.39	1.44	1.39	1.36	1.51	1.46	1.76
20	2.33	2.28	2.43	2.44	2.40	2.36	2.52	2.41	2.11	2.10	2.10	2.09	2.11	2.20	2.39	2.39

**Figure 3:**
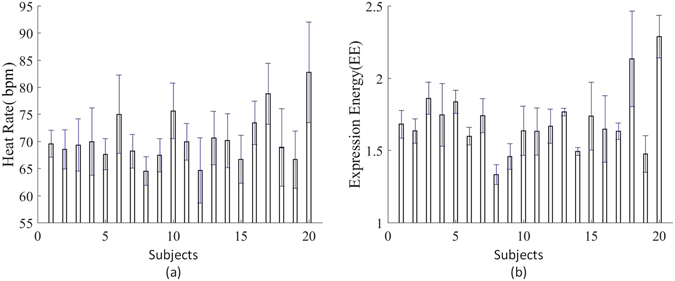
Individual difference in HRs and EEs. Each bar represents the average for the 20 subjects for HRs and EEs. Error bars represent standard deviations (S.D.).

Next, the location of the ROI, within each frame, was obtained which allowed for the extraction of the latent HR information. The bpm data, also at 10 second intervals, are tabulated in Table 2. Figure 3 illustrates the means and the standard deviations of HRs and EEs for each subject. Analysis of variance (ANOVA) further shows significant individual differences in HRs and EEs while viewing the video stimuli (*ρ* < 0.001, *F* = 12.95 and *ρ* < 0.001, *F* = 34.72).

**Table 2:** The bpm of 20 subjects down-sampled to 10 second intervals for presentation. The variation of HR measurement is still clear for all subjects at ten second of time interval.

Subjects	Time Interval(second)															
	10	20	30	40	50	60	70	80	90	100	110	120	130	140	150	160
1	66	70	70	68	68	69	67	70	70	68	69	74	70	68	71	76
2	72	70	70	64	72	66	67	69	70	70	67	71	70	69	58	72
3	68	73	62	66	75	70	69	73	70	71	69	68	75	75	57	69
4	65	82	60	58	75	66	73	66	75	71	69	77	69	70	75	69
5	65	65	66	69	68	74	67	64	66	68	65	70	67	72	71	66
6	70	82	76	63	76	75	76	65	84	86	70	85	77	74	64	78
7	67	72	66	62	68	72	68	71	73	65	64	70	67	69	71	67
8	65	67	62	66	64	68	61	61	67	62	66	62	64	70	63	65
9	73	68	65	69	64	64	69	73	66	68	65	72	68	65	65	66
10	82	73	78	71	79	81	83	74	73	78	73	84	73	72	66	71
11	70	74	69	68	66	71	69	65	74	73	75	71	71	73	64	67
12	72	56	75	66	60	65	62	59	65	68	70	65	71	69	56	56
13	72	79	73	68	66	57	71	73	72	72	73	76	72	72	66	69
14	67	71	70	67	57	69	70	68	77	73	68	75	70	68	75	78
15	63	67	69	62	63	71	68	70	65	69	68	69	72	72	65	55
16	78	77	72	67	71	67	74	72	74	74	69	81	72	75	74	79
17	73	80	79	64	79	76	79	80	88	83	77	83	75	87	77	81
18	58	78	68	72	74	62	57	62	81	72	62	75	68	72	67	75
19	56	67	61	68	65	68	64	70	66	71	65	71	64	74	60	77
20	79	82	66	67	81	82	83	80	94	92	92	97	94	76	75	85

### Non-parametric joint density estimation in SAS

**Figure 4:**
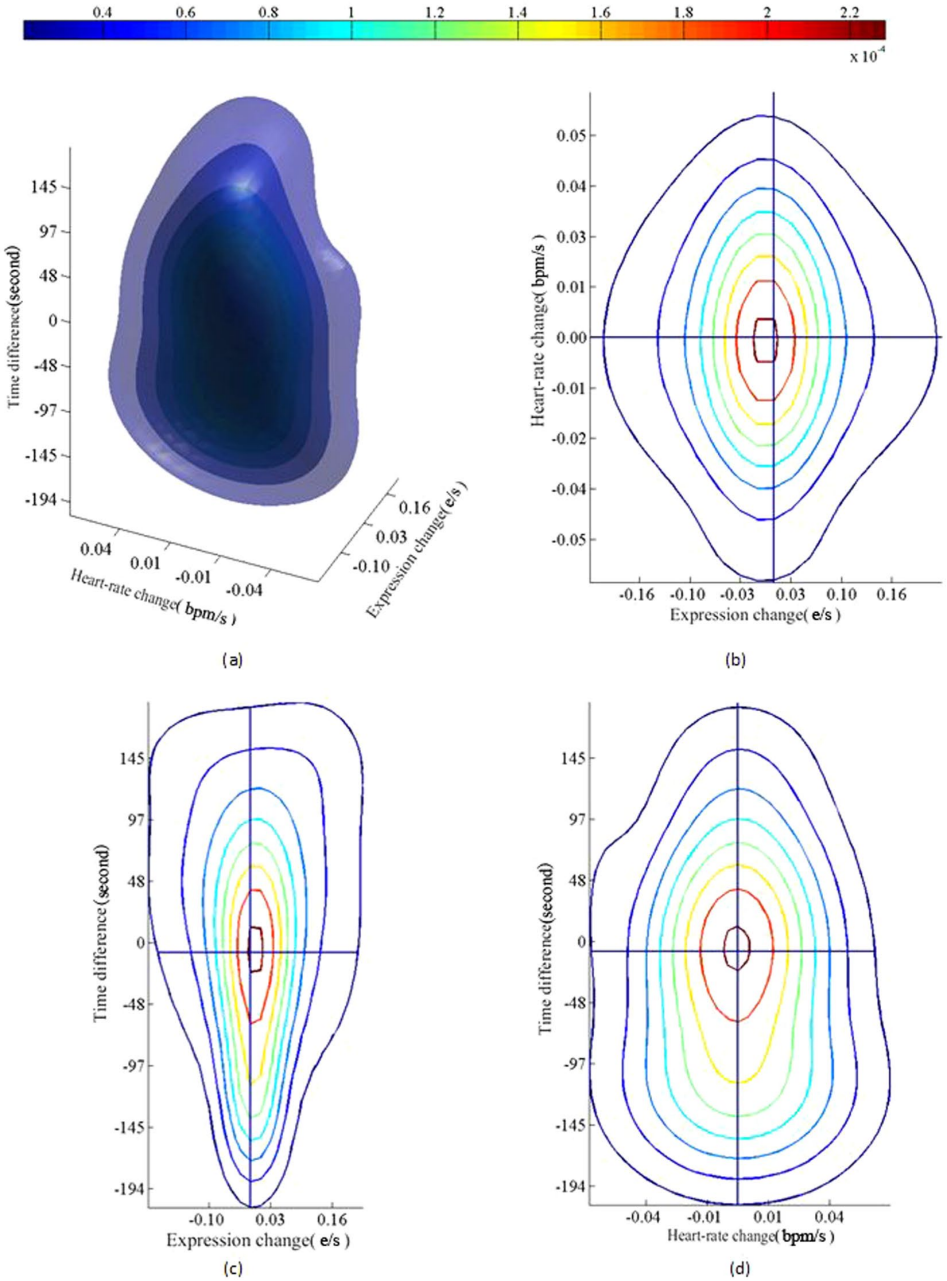
Density map of 3D SAS and its sectional views. Different colors denote different isosurfaces (i.e. the same density value). The three dimensions of SAS are measured respectively per second, the unit “bpm/s” and the unit “E/s”, wherein the character “*E*” is the shortened form of EE defined in Equation (2).

The physiological changes and spontaneous expressions were sampled, at the frames with large variation, into the Statistical Association Space (SAS) by using the pairing equation, Equation (3) with a 160 s time window. A Gaussian Mixture Model (GMM) was used to generate the probability density maps, such as is shown in Figure 4(a). The *XY*, *XZ* and *YZ* section views of the map are shown, respectively, in Figure 4(b), (c) and (d), with *X* and *Y* corresponding to *changes* between successive samples of the Expression Energy and HR, respectively, and *Z* is the difference in time between paired events.

The probability density map in Figure 4(a) presents a typical distribution, descending gradually in density from its center to the outside. Its form may be used to reveal a number of characteristics of the dynamic correlation between the two cues of physiological changes and spontaneous expressions. For example, from the contour values on the slice at zero time-difference (Figure 4(b)) one can assess the degree to which peaks in the two cues are coincident.

**Figure 5:**
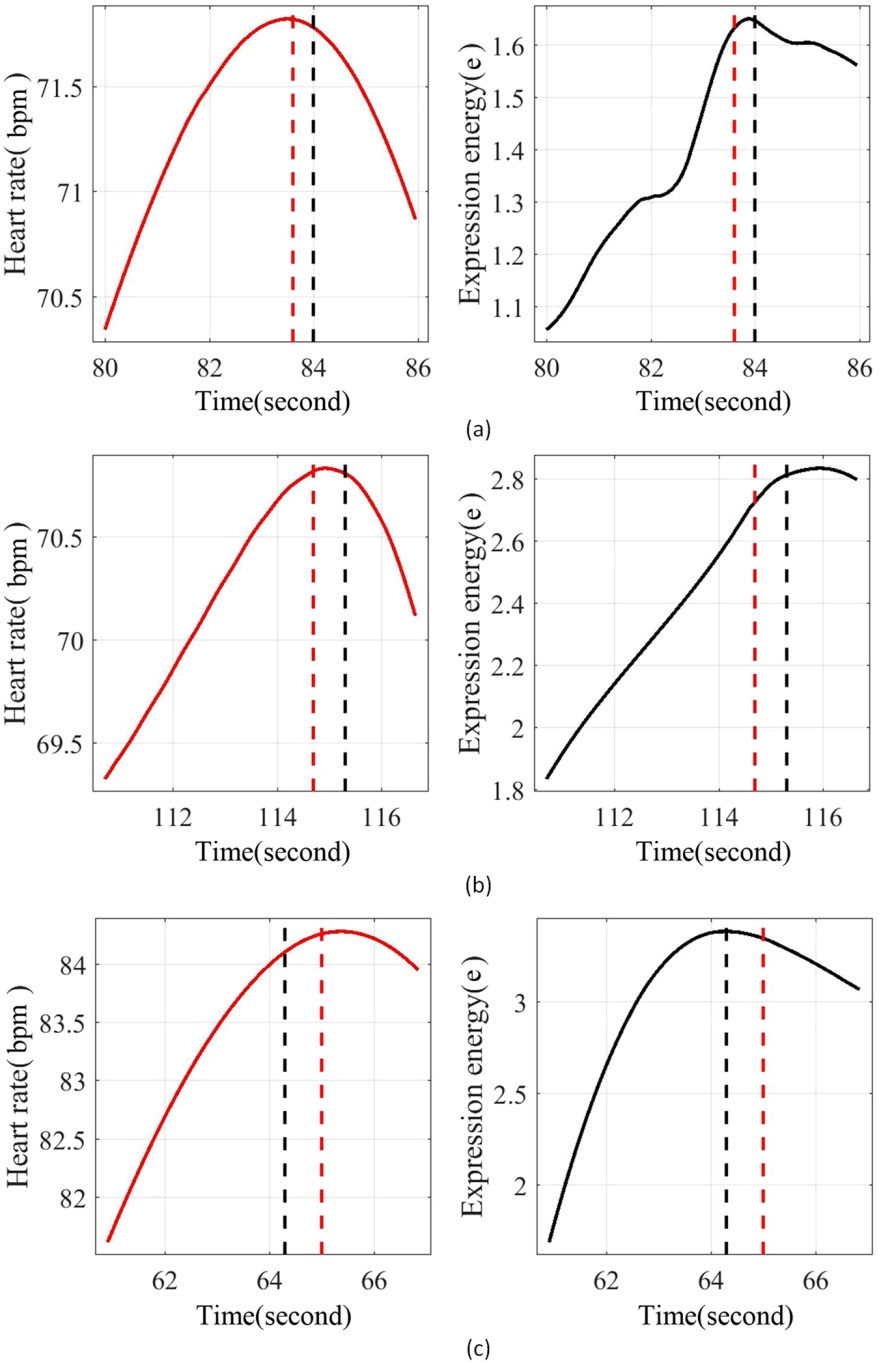
The correlation examples from three subjects. These examples are chosen from the high density region defined by [−0.03,0.03] × [−0.01,0.01] × [−1.0,1.0] of 3D probability map. The red and black dashed lines respectively denote the temporal positions where the correlation occurred.

The correlation examples from three of the subjects are shown in Figure 5. These examples were sampled in the high density region, [−0.03,0.03] × [−0.01,0.01] × [−1.0,1.0], of the 3D probability map to give a picture of the dynamic interaction of two cues. The vertical dashed lines denote the times of paired HR (red) and EE (black) events.

### Wavelet coherence analysis

**Figure 6:**
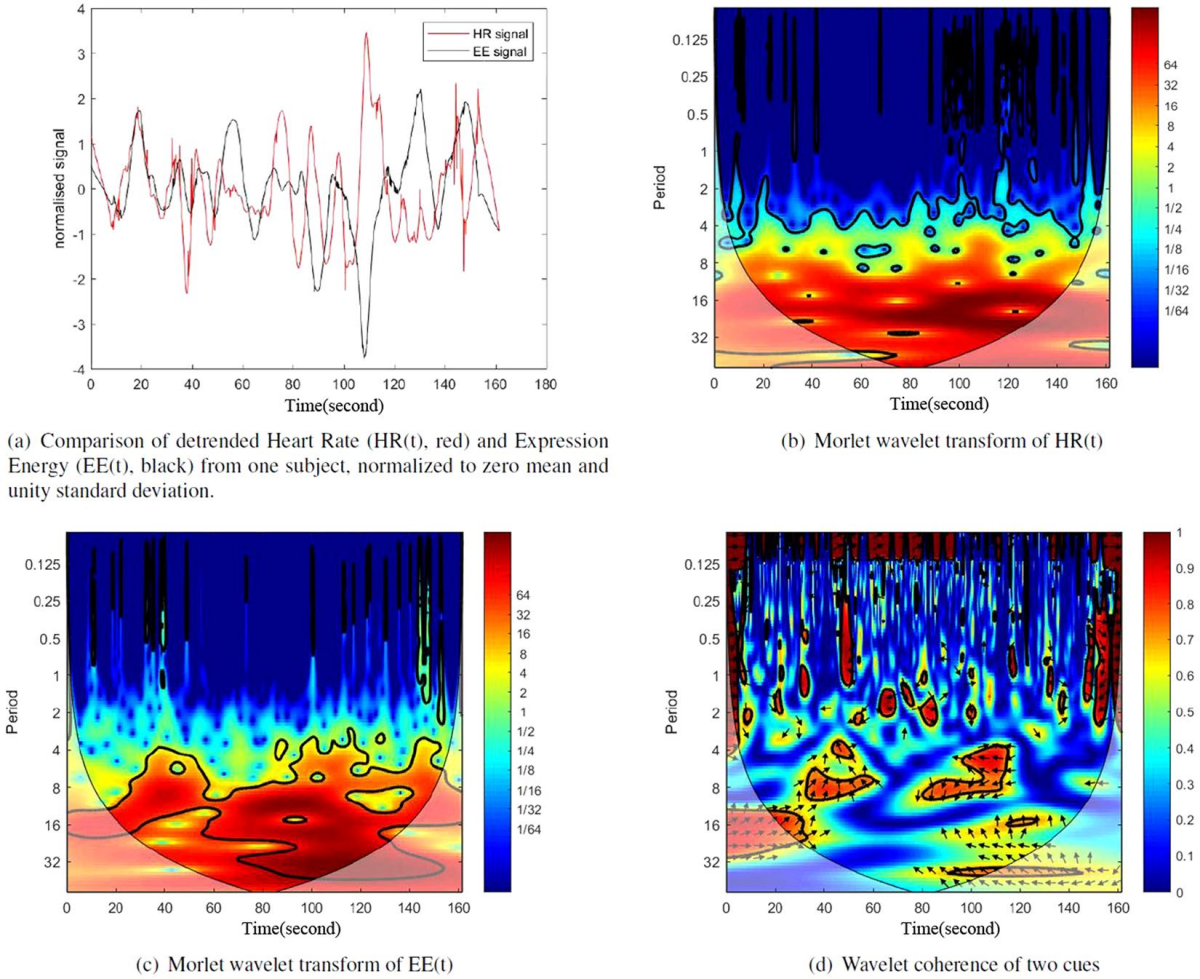
Wavelet coherence analysis of the example pair of HR(t) and EE(t) signals shown in (**a**).

The proceeding analysis was used to examine the correlation in time-frequency space between the two cues from each subject. The peak values of two cues (rather than their derivatives) also showed similarities under Morlet wavelet analysis, but there were delays between these peak effects that varied throughout the video sequence. Figure 6 demonstrates an example of wavelet coherence analysis (WCA) of an HR and EE series. Figure 6(a) shows the two cues (detrended and normalized to zero mean and unity standard deviation) from one subject which clearly demonstrates a change in the time delay between peak values throughout the video sequence. Figure 6(b) and (c) respectively show a Morlet wavelet transforms of the HR and EE signals shown in Figure 6(a). In these two figures, the majority of the energy exists in the same frequency range but is seen to occur at different times (dark red regions). The correlations between the wavelet transforms can be seen through the wavelet coherence plot^35^ shown in Figure 6(d), where the arrows indicate the phase lead or lag and the time differences.

## Discussion

This study explores the dynamic correlation between physiological changes and spontaneous expressions, as acquired by stereo vision based tracking and iPPG. The HR and EE series obtained for each subject, resampled at 10 second intervals, are tabulated in Table 1 and Table 2, and their means and standard deviations are illustrated in Figure 3. ANOVA tests revealed significant differences within an individual’s HRs and EEs whilst viewing their video stimuli (*ρ* < 0.001, *F* = 12.95 and *ρ* < 0.001, *F* = 34.72), due to the influence of individual physiological and mental characteristics, as well as the different video content. These individual differences in HR and EE, also differed between subjects. As a result, we study here the dynamic correlation between physiological changes and spontaneous expressions by focusing on signal changes which are essentially represented by derivatives.

Seeking a general rule for the association of physiological changes and spontaneous expressions, this study has attempted to reduce the bias towards certain emotional categories through emotion induction. For this, a wide diversity of induced emotions in the stimuli selection was ensured; randomized inputs were used and their use limited when presenting stimulus videos to the subjects. This also helped to avoid artifact correlations resulting from particular story-lines in the stimulus videos.

Non-parametric joint probability density estimation in the SAS revealed several characteristics of the dynamic correlation between peaks in HRs and paired peaks in Expression Energy (see Figure 4). 1) The two cues tend to have a tighter associations when in their peak states. This is clear as the probability density is centered at the (X,Y) origin, indicating two cues are each close to a peak. 2) The cumulative distribution on the surface of zero time-difference is smaller than 0.047 so that this slice represents a very small contribution to the overall probability. This also suggests that the two cues barely correlate at all when considered as simultaneous in time. 3) The shape of the overall probability density indicates that correlation between the two cues is likely to be asynchronous and complex (nonlinear). In brief, the two cues have a close interaction, but in an asynchronous and a nonlinear way, in which the peak of one cue heralds a peak in the other.

The Wavelet Coherence Analysis (WCA) was also used for these two cues to examine the dynamic correlation in the localized oscillations, and it produced similar results. The appearance of phase leads and lags in the coherence plot, between HRs and EEs, also proved the correlation of the peak values of two cues, but with delays between the peaks that varied throughout the video sequences. These results are consistent with the non-parametric joint density estimation used to reveal the dynamical connections between cues. The consistency gives a confidence to the results, and of the validity of non-parametric joint density estimation in the SAS.

The WCA is able to find oscillatory patterns which possess a dynamic correlation between the two cues. Unfortunately, its application is limited to cases with fairly well-aligned cues. In this study, this was only applied to the HR and EE series from one subject. Non-parametric joint density estimation in the SAS was performed on HR and EE signals from different subjects by sampling at points of large local changes in the time window, to accurately locate the onset of local changes in the cues. More importantly, this may reveal a statistical correlation tendency across different subjects.

The tendency that two cues have a tighter association when they are approaching peak states, suggests a regulatory procedure. Whatever modulates the physiological and mental activities to form this tendency has not been previously described, and recognizing this tendency represents a first step in understanding this regulatory procedure.

Three correlation examples (as shown in Figure 5) also show there are significant difference in HRs and EEs between individuals while viewing video stimuli. In these examples, it can be observed that the individual differences in EEs is much larger than those in HRs. ANOVA testing agrees with this observation (*F* = 12.95 for HRs and *F* = 34.72 for EEs). In examples of 1 and 2, the fluctuations in EE as they reach their peak are of a different size from those observed in the HRs. In the example 3, the EE rises rapidly and smoothly to its peaks, in advance of the associated peaks in HR. The larger individual difference in expression shows more complex influence factors exist in the expression procedure.

An additional aspect of this study provides an empirical interpretation of a long-time-span correlation^19^. The method of randomization and limitation when presenting the stimulus videos conveys a “pure” density map in which the high density region corresponds to a small, rather than a large, time-difference. This supports the observation that long-time-span correlations reflect the storyline correlation.

A further study will be carried out to analyze the influence factors on expression, and to take into account expression labels and dimensional descriptions, and other modalities of expression or physiological cue. The correlation patterns among these modalities will be further explored, and their contributions to affection judgment will be assessed in detail.

## Methods

**Figure 7:**
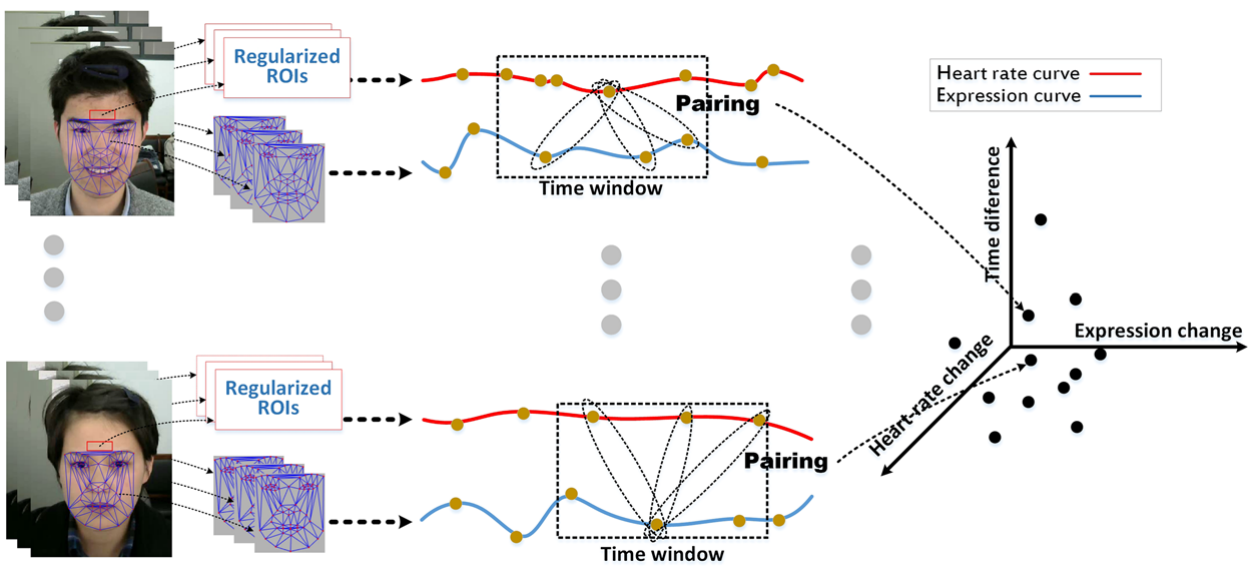
Overview of the method composed of tracking, pairing and evaluation.

Figure 7 presents a schematic overview of the method of tracking, pairing and evaluation. The HRs was obtained by iPPG under controlled room conditions (Temperature: 18 ± 2*C*°, Humidity: 50 ± 1%) with sufficient natural ambient illumination. The validation of HRs against iPPG was carried in the same conditions by measuring an EEG signal with a three-lead electrocardiogram system (ML846 PowerLab 4/26, ADInstruments, Australia).

### Stereo vision based tracking

Through two synchronized cameras, a sequence of coupled, simultaneous frames were captured to record the instantaneous expressions of a subject’s face. By using a tree-structured model^36^. the 2D positions of the 66 facial landmarks were detected respectively on each couple of simultaneous frames, then fed into a stereo vision analysis package^24^ to retrieve the 3D positions of the facial landmarks for the accurate depiction of the instantaneous facial expressions.

With the 3D positions of the facial landmarks, an affine transformation can be accurately determined within each frame, so that the ROI may be fixed. Essentially the coordinate axes for the region of interest (ROI) are obtained by finding the symmetry plane^37^ *P* of the landmark positions for the eyes, eyebrows and nose, and a second plane *P*′ perpendicular to *P*. *P*′ is then moved, perpendicular to *P*, until it is closest to the inner corners of the two eyebrows (*e*_1_ and *e*_2_), in a least squares sense. In this way, the facial mid-line *M* for the frame (in plane *P*), and the perpendicular line *M*′ (formed from the intersection of *P* and *P*′) define the vertical and horizontal axes for the ROI. This then defines the affine-invariant geometrical relationship which fixes the ROI in the current frame.

### Opto-physiological modeling driven imaging photoplethysmography

The concept underlying iPPG is an opto-physiological model (OPM) which here is determined in the context of a reflection-mode system. The physical assumption implicit in PPG is that changes in measured light intensity are due to changes in blood volume. Light transmitted through the anatomy of the subject, results in a measured intensity that depends upon the wavelength and intensity of source in addition to the optical interaction with the subject. The interaction of light trans-illumination can be expressed through the Beer-Lambert law^25^, which defines the light transmittance through a medium such as blood, tissue, bone, etc., in terms of its molar coefficient of absorption and the length of the optical path *l*.

1 $$I(\lambda )={I}_{0}\exp \,(-\mu (\lambda )l)$$

where *l* is the optical path length, *I*_0_ is the source intensity, and *μ*(*λ*) and *I*(*λ*) are the extinction coefficient and received intensity for a light source of wavelength *λ*.

### Statistical Association spaces (SAS)

We denote by *p*_*k*_(*t*), the 3D position of the *k* ^*th*^ landmark in frame *t*. The Expression Energy *E*(*t*) for that frame is then determined from these positions as the sum of the square relative displacements, (*p*_*i*_(*t*) − *p*_*j*_(*t*))^2^ over the set (*i*,*j*) ∈ *P*_*E*_(*t*) (corresponding to those landmarks associated with the eyes and eyebrows) and the set (*n*,*m*) ∈ *P*_*M*_(*t*) (corresponding to those landmarks associated with the mouth and nose). Thus we define:

2 $$E(t)=\sum _{(i,j)\in {P}_{E}}{({p}_{i}-{p}_{j})}^{2}+\sum _{(n,m)\in {P}_{M}}{({p}_{n}-{p}_{m})}^{2}$$

From each frame of an expression video sequence an expression energy value, *E*(*t*), is retrieved. The retrieved EE sequence is then fitted with a continuous curve *c*_*E*_ by the Lowess Smoothing algorithm^33, 34^ which conducts a local regression using weighted linear least squares, in which outliers in the sequence are reduced in influence by assigning a lower weight.

Also, by the means of the Lowess Smoothing algorithm, another continuous curve *c*_*H*_ is acquired from the related HR sequence which was extracted from an expression video through the following steps. First, a regularized ROI in each frame is divided into a discrete set of subwindows to produce a new set of reduced ROIs, where the value of each pixel in the reduced ROI is set as the average of all the pixel values within its subwindow. Though compromising the spatial resolution, such a procedure is applied to significantly improve the signal-to-noise ratio. In the present study, the subwindow size was set to 8 × 8 pixels. This resulted in a reduced ROI size of 2×4 pixels, yielding raw iPPG signals at each pixel position across a sequence of frames. The iPPG signals were then bandpass filtered with a fifth-order Butterworth filter with cutoff frequencies set at [0.5, 4] Hz. A joint time-frequency analysis was then performed on the iPPG signals to reveal the time-varying HRs via a short-time Fourier transform (STFT)^38^.

The local variation in the intensities of two cues are measured as *v* = |(*x*−*μ*)/*μ*|, where *x* is the mid-value of a two-second segment of the curve *c*_*E*_ or *c*_*H*_, and *μ* is the mean of the segment. Through the following pairing function, sampling was then performed between the points at the frames with a large variation (*v* > 3%):

3 $$s({\upsilon }_{t,E},{\upsilon }_{t^{\prime} ,H},t^{\prime} -t)=pairin{g}_{|t^{\prime} -t| < W\mathrm{/2}}({e}_{t,E},{e}_{t^{\prime} ,H})$$

where *e*_*t*,*E*_ is a point of expression curve *c*_*E*_ at frame *t*, *e*_*t*′,*H*_ is a point of physiological curve *c*_*H*_ at frame *t*′, and *W* is defined as a time window for pairing. The returned value *s*(*υ*_*t*,*E*_,*υ*_*t*′,*H*_,*t*′ − *t*) of the pairing function is the joint representation of a sampled point, where *υ*_*t*,*E*_ is the derivative of continuous curve *c*_*E*_ at frame *t*, and *υ*_*t*′,*H*_ is the derivative of continuous curve *c*_*H*_ at frame *t*′. Here, the first order derivatives of continuous curves *c*_*E*_ and *c*_*H*_ are used to represent local changes or oscillations of physiological and expression energy series. This forms a 3D statistical association space (SAS) of expression change, physiological change, and their time-difference.

The probability density distribution of 3D association space was computed by use of a Gaussian Mixture Model (GMM) to interpret the dynamic correlation between physiological changes and spontaneous expressions.

### Stimuli selection

Selecting the most effective stimuli is crucial to elicit spontaneous expressions from the selected subjects. To minimize the bias from a manual stimulus selection, a semi-automated method was used. The stimuli were short audiovisual video clips, which were selected from those listed on these well-known video websites, including Tudou, Ku6, iQiyi, Youku and Letv. These websites allow users to give comments and assign tags to the individual video clips. Many of the tags carried emotional meanings, such as exciting or aggressive.

For each of the emotional keywords taken in the study^39^, the video clips with the corresponding tags were found from the above websites, and graded by corresponding tag numbers. The top 3~5 video clips corresponding to each emotional keyword were selected initially which resulted in a total of 367 video clips.

In order to ensure diversity of induced emotions, from the 367 video clips, a final set of 35 video clips with the lengths 53~57 seconds was finally selected based upon the following criteria:

1. Is the tag consistent with the affective content?
  Some video clips were subjectively refused because they were merely tagged by their title, artist name or lyrics of incidental music, but where their actual emotional content was entirely different (e.g. sad video clips with happy topics).
2. Is the video clip a good fit for utilization in the experimental protocol?

The subjects in the implementation of the experimental protocol were mostly young students. The stimuli selection focused on the video clips which were most likely to elicit emotions for this target demographic.

### Experiment setup and validation procedure

Parameters relating to the experimental setup are listed in Table 3, with its layout displayed in Figure 1. The tests were taken in a controlled laboratory environment (Temperature: 18 ± 2*C*°, Humidity: 50 ± 1%), with natural ambient illumination (>2300LUX), during the middle of the day (11:00 to 13:00). The cameras were connected through two trigger lines and two USB cables to a PC (ThinkCentre M8600T, Lenovo) which recorded the facial videos. The video stimuli were presented to subjects using the professional experimental design software E-Prime^40, 41^, installed on a laptop (P4 3.3GHz, Lenovo), as presented in Figure 1. E-prime also was used to ensure synchronization by sending the synchronous mark to the PC, which forwarded it immediately to the two digital cameras.

**Table 3:** Experimental setup to acquire spontaneous facial expression videos.

Experimental Setup	Parameter/Details
Two Cameras	Model: MV-UB 130 GC, MindVision, Shenzhen, China. 1.3 Megapixel, and Standard C-mount lens, global shutter, hardware trigger.
Lens	3 Megapixel 1\ 2 6–12mm F1.6-C, Guangzhou, China.
Distance between two cameras (mm)	300mm
Subject and camera (mm)	550–650mm
Frame rate (fps)	30
Software used	E-Prime

The HR measurement in the above experimental setup was validated by the three-lead ECG system (ML846 PowerLab 4/26, ADInstruments, Australia). Together with the two digital cameras, the ECG system was also connected to a recording PC, which was started by the synchronous marker pulse from the stimulus laptop. Thus the HR reading from a subject was simultaneously captured by the ECG system and the two camera iPPG setup. The validation was conducted, in a separate initial experiment, by comparing the HR cues respectively from the ECG and the iPPG.

### Implementation of experimental protocol

**Figure 8:**
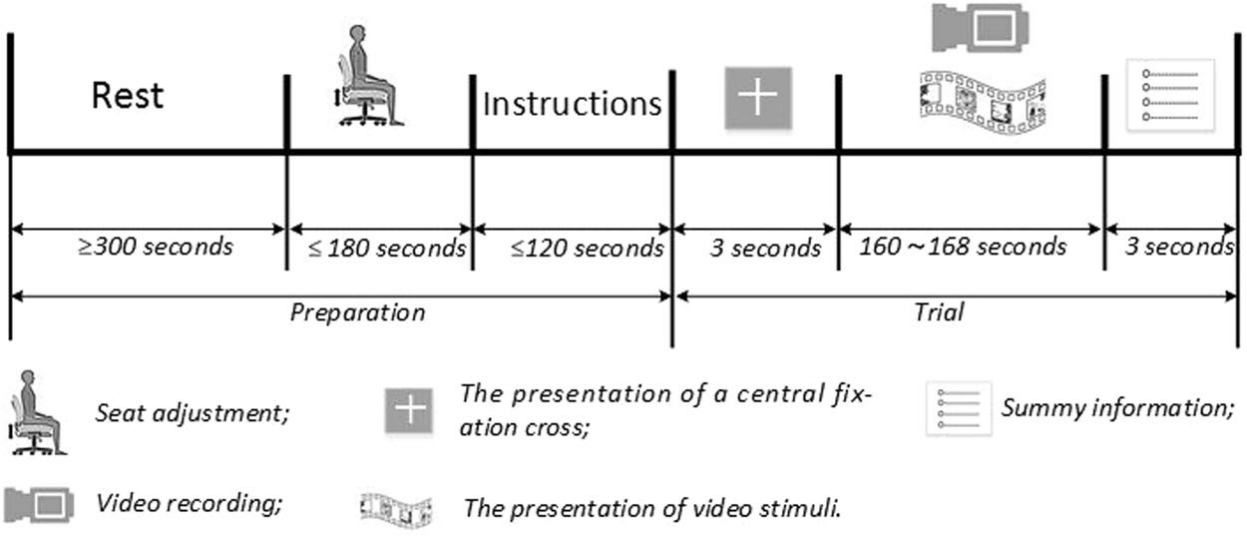
A schematic diagram of the experimental protocol procedures.

The procedure for the experimental protocol are depicted in Figure 8. Twenty healthy subjects (Gender: 10 males and 10 females; Age: 20 to 25) who gave written informed consent, were invited to participate in the implementation of the experimental protocol. The study was approved by the Ethics Committee of Shanghai University and performed in accordance with the Declaration of Helsinki.

Prior to the implementation of the protocol, each subject was required to rest, for at least five minutes, before being guided to the test room. When sitting in front of a laptop, the subject had his/her seat adjusted carefully with the assistance of the experimental staff to avoid myogenic artifacts arising from neck and temple muscles. Then the subject was given the prepared instructions, together with the tasks required to implement the protocol. Once the subject was satisfied with the instructions, the experimenters left the test room.

The subject was able to start the trial by pressing any key on the keyboard. Each trial began with a presentation of a central fixation cross on a gray background for three seconds. Then three of the 35 video clips corresponding to the different emotional keywords were chosen and played randomly by the E-Prime software. E-Prime was programmed to ensure each video clip would be presented just once to a limited number of subjects. In this study, the limit number was set just to two in order to ensure that only a small number of the 20 subjects could watch the same video clips. Additionally, E-Prime was responsible for synchronizing the recording PC by sending the synchronous marks when playing the video stimuli. The above randomization and limitation in the presenting stimuli was performed to reduce the bias towards any single emotional category in emotion induction, and to avoid artifact correlations resulting from the story-lines in the video stimuli^19^. When the video sequences finished, the E-Prime program stopped recording and displayed the summary information including time consumption, video clip names and date. The next trial could be started by pressing any key again. Finally 20 trials with 20 subjects were obtained from the 35 video clips.

### Artifact reduction

In the implementation of the experimental protocol, the controlled environment was used to eliminate unnecessary interference from the surroundings. Two high-performance digital cameras were used to ensure the accuracy of the stereo vision based tracking, and to reduce the possibility of artifacts arising in physiology and expression measurements through, for example, use of inaccurate 3D positions of the facial landmarks. Similarly the preparation steps avoided artifacts from the emotional state of a subject prior to the trial. False correlations were also limited by the randomized and limited presentation which reduced any imbalance in the emotion categories, or storyline correlation in video contents as described in the Discussion section.

In the emotion induction, the spontaneous emotional responses of subjects, when viewing the video stimuli, were occasionally accompanied by head movements. This created a larger motion in the facial images captured by the two cameras. However, through the stereo vision based tracking, the expression measurement was largely unaffected by this motion. Moreover, the physiological measurement was able to be conducted using a consistent and nearly-motionless forehead region with any head motion artifacts being much reduced.

As a rectangular ROI might be mapped into different four-sided regions on each frame due to projection variations, it was necessary to regularize the mapped ROIs by a piecewise affine warp. Such a regularization procedure allows a right alignment of ROIs across sequential frames and reduced the effect of possible projection artifacts allowing a good readout of physiological changes.

The Lowess Smoothing algorithm was an additional step used to reduce artifacts mixed in with the obtained EEs and HRs. Such artifacts represent as outliers, mainly caused by occasional large errors in the facial landmark detection.

In each stage of the proposed experiment, the main artifact sources were carefully considered and corresponding suppression steps was taken. Through the above multiple suppression activities, the various artifacts in this study were intercepted at an early stage, and thus did not cause any systematic issues. Any artifacts surviving into the SAS were individual, isolated and accidental, and would display with a low density. Consequently by using a probability density estimation in the SAS, an effective evaluation of the correlation between physiological changes and spontaneous expressions was able to be achieved.
